# Association between cardiopulmonary function, health-related quality of life and cognitive impairment among the older nursing home residents in Shanghai, China

**DOI:** 10.1017/S1463423623000075

**Published:** 2023-03-15

**Authors:** Zhitong Zhou, Longbing Ren, Ziyan Zhang, Xiaoting Sun, Yongtao Zheng, Yijun Gu, Hengjing Wu, Jue Li, Lijuan Zhang

**Affiliations:** 1 Clinical Center for Intelligent Rehabilitation Research, Shanghai YangZhi Rehabilitation Hospital (Shanghai Sunshine Rehabilitation Center), Tongji University School of Medicine, Tongji University, Shanghai 201613, China; 2 Institute of Clinical Epidemiology and Evidence-based medicine, Tongji University School of Medicine, Shanghai 200092, China; 3 Tenth People’s Hospital Affiliated to Tongji University, Shanghai 200072, China; 4 Xiaoshan Center for Disease Control and Prevention, Hangzhou 311200, China; 5 The Second Affiliated Hospital of Zhejiang University, Hangzhou 310009, China; 6 Department of Epidemiology, Tongji Hospital Affiliated to Tongji University School of Medicine, Shanghai 200065, China

**Keywords:** cognitive impairment, health-related quality of life, left ventricular ejection fractions, vital capacity

## Abstract

**Background:**

This study aimed to examine the association between cardiopulmonary function, health-related quality of life (HRQOL) and cognitive function among nursing home residents aged 80 years and over.

**Methods:**

A nursing home-based, cross-sectional study was implemented among 677 aged over 80 years in Shanghai, China. A total of 197 participants underwent effective cardiopulmonary function examinations. Mini-Mental Status Examination (MMSE) and Short Form-36 scales (SF-36) were used to assess cognitive function and HRQOL, respectively.

**Results:**

Decline in left ventricular ejection fractions (LVEF) [adjusted odds ratio (AOR), 1.98; 95% confidential interval (CI), 1.03–3.81)] and vital capacity (VC) (AOR, 2.08; 95%CI, 1.07–4.04) was associated with cognitive impairment. After adjusting confounding factors, relationships between cognitive function and physical functioning (PF) (AOR, 0.98; 95%CI, 0.97–0.99) still existed.

**Conclusions:**

Healthcare professionals should pay more attention to cardiopulmonary health and HRQOL in the nursing home residents. Actions of public health strategies focus on the improvement of cardiopulmonary function, and PF among older nursing home residents with cognitive impairment is required.

## Introduction

Approximately 9% of the global population is aged 65 or over in 2019, predicted to increase to 16% by 2050 (Qiu *et al.*, 2022). As the population ageing, age-associated diseases and disabilities worldwide become an important public health issue, which imposes heavy economic burden on society and families (Pérez Palmer *et al.*, 2022; Su *et al.*, 2014). Cognitive impairment has tended to worsen to a great extent in recent years. The estimated prevalence of cognitive impairment is 10%–15% among population over 65 years and reaches 25% in those aged 80–84 (Anderson, 2019; Pérez Palmer *et al.*, 2022). Population aged 80 years and over comprises the fastest-growing segment of Chinese population. The prevalence of cognitive impairment ranges between 6% and 16% among Chinese people aged 60 years and over (Jia *et al.*, 2020) and reaches around 48% in people aged more than 80 years old (An and Liu, 2016). Primary health care plays a vital role in cognitive impairment (Mullins *et al.*, 2021). Nevertheless, current primary health care faces serious challenge in preventing and managing cognitive impairment in the elderly population (Belmin *et al.*, 2012; Lu *et al.*, 2022).

Due to lack of family companionship, cognitive function of nursing home residents is more worthy of attention. Notably, the prevalence of CI in the older nursing home residents is higher than the community elderly (Wu *et al.*, 1998; Miranda *et al.*, 2021; Yuan *et al.*, 2021; Qiu *et al.*, 2022). Previous studies report age, married status, male, diabetes, low income, low education, unfavourable cardiovascular health and carrying apolipoprotein E (APOE) e4 allele were risk factors of cognitive impairment (Peloso *et al.*, 2020; Frison *et al.*, 2021; Najar *et al.*, 2021). Cognitive impairment is always accompanied by declines in health-related quality of life (HRQOL), disability, death and cardiopulmonary dysfunction. About 18% of CI patients experience respiratory dysfunction (Martinez *et al.*, 2014), and 43% of cognitive decline patients have heart failure (HF) (Cannon *et al.*, 2017). Accumulating evidence from different countries not only indicates abnormal cardiopulmonary function increased the risk of cognitive impairment (Emery *et al.*, 2012; Cermakova *et al.*, 2015; Jefferson *et al.*, 2015; Zhang *et al.*, 2020; Duong *et al.*, 2022) but also reveals the complex interactions between cognitive impairment and HRQOL (Ezzati *et al.*, 2019; Liu *et al.*, 2019; Phyo *et al.*, 2021). Currently, the mechanisms of abnormal cardiac function and cognitive impairment mainly involve cerebral hypoperfusion, hypoxia, systemic inflammation, endothelial injury and neurohormonal activation (Cermakova *et al.*, 2015; Diener *et al.*, 2019) and of abnormal pulmonary function and cognitive impairment mainly involved hypoxia, systemic inflammation and cerebral vascular dysfunction (Wang *et al.*, 2020). Related studies mainly focus on community populations under the age of 80. The cognitive function of order nursing home residents received less attention. However, the association between abnormal cardiopulmonary function, HRQOL and cognitive impairment remains to be clarified in the nursing home residents aged over 80 years old.

In this study, we aimed to assess the association between cognitive impairment and HRQOL and cardiopulmonary function, so as to explore the notable indicators of cognitive impairment, therapy providing evidence for the prevention and control of cardiovascular-related cognitive dysfunction and reduction of the disease burden of cognitive impairment.

## Methods

### Study participants

Strategy of participants’ selection using a multi-stage stratified cluster sampling method in this study is shown in Supplementary material 1. Briefly, a randomized district in the urban and suburban areas of Shanghai from 2016 to 2017 was selected, respectively, and then randomly selected four nursing homes from each district, and a total of 677 participants who met the criterion were recruited in the study.

Participants enrolled in the study met the following conditions: (1) participants were at least 80 years old; (2) locally residents lived in nursing homes in Shanghai; (3) had normal hearing, vision and speech.

Exclusion criteria included (1) absence of cognitive function or in a vegetative state; (2) diagnoses of schizophrenia or serious mental retardation; (3) suffered a traumatic brain injury; and (4) inability to cooperate with the following inspections or assessment.

Each participant received physical and chemical inspection, face-to-face interview and cognitive assessment.

For effective assessment in cardiopulmonary function and HRQOL, the additional exclusion criteria were formulated: (1) unable to follow the precautions for cardiopulmonary function tests as directed by the researchers; (2) patients who could not complete the questions about daily activities in HRQOL test. Finally, a total of 197 participants who met the criteria were included in the analysis.

### Cognitive assessment

Chinese version of the Mini-Mental State Examination (MMSE) was used to evaluate cognitive function because of the high proportion of low-educated subjects in this study (Wu *et al.*, 2021). The MMSE is a 30-point questionnaire assessing five areas of cognitive function including orientation, registration, language and praxis, attention and calculation, and recall, and education attainments were adjusted when calculating the raw total score. Cognitive impairment was classified if MMSE ≤ 17 for illiterates; MMSE ≤ 20 for elementary school graduates; MMSE ≤ 24 for junior school graduates or above (Li *et al.*, 2016). Cronbach’s alpha value of the MMSE scale was tested to examine the reliability of the MMSE questionnaire, and the result was 0.899, indicating that it can be utilized to assess cognitive impairment of subjects in this study.

### Assessment of HRQOL

Chinese version of the Short Form-36 scales (SF-36) was used to measure HRQOL of participants, which not only has a better split-half reliability (*r* = 0.91, *P* < 0.001) (Zhou et al., 2018) but also is a valid and reliable tool for assessing HRQOL among Chinese population (Wang *et al.*, 2008). SF-36 includes eight scales, evaluating the following areas: physical functioning (PF), role limitations due to physical health (RP), body pain (BP), general health (GH), vitality (VT), social functioning (SF), role limitations due to emotional problems (RE) and mental health (MH). Cronbach’s alpha value of Chinese version SF-36 scale was 0.821 in this study, suggesting good internal consistency of the scale.

### Assessment of heart function

Heart function was measured by trained technicians via the Vscan V1.2 (GE Healthcare, Milwaukee, WI, USA). The sensitivity and specificity of palm ultrasound to detect left ventricular dilatation range from 71% to 94% and 97% to 100%, respectively (*Liebo et al.*, 2011). According to the American Society of Echocardiography (ASE) (Lang et al., 2005), heart rate, systolic blood pressure (SBP), diastolic blood pressure (DBP), pulse pressure (PP), end-diastolic dimension of left ventricle (LVD), end-systolic dimension of left ventricle (LVS), the thickness of the basal interventricular septum (IVS), left ventricular posterior wall (LVPW), LVEF and calculated left ventricle fractional shortening (LVFS) were determined. The measured values were divided into normal and abnormal groups according to the standard (Supplementary material 2) (Yancy *et al.*, 2017).

### Assessment of respiratory function

Respiratory function was measured using CHESTHI-101 (COSMED S.r.l. Japan). Vital capacity (VC) is the amount of air that people can try to exhale after maximal inspiration. Maximal ventilatory volume (MVV) was collected to measure elasticity of thoracic lung tissue, airway resistance and respiratory muscle strength. However, forced expiratory volume in 1 s, which is a commonly used indicator to determine asthma and chronic obstructive pulmonary disease, was not collected because it is difficult for ultra-aged people to exhale in a second.

### Other baseline measurements

Sociodemographic information and health-related data were collected for all participants by using an interviewer-administered questionnaire, including age, gender, education, marital status, smoking status, body mass index (BMI, weight, in kilograms/height^2^, in metres), physical activity, disease histories and medication histories. Education attainment was divided into 3 groups: illiteracy, elementary school (<6 years of education) and junior school or above (≥6 years of education). Marital status included unmarried, married, widowed and divorced, and we further defined it as a dichotomous variable (with and without partners). Physical activity was defined as standing, walking and outdoor time of participants (≤1 h/>1 h). Information of disease history including previous hypertension, diabetes, dyslipidemia, coronary heart disease, stroke and tumour were collected by self-reported physican's diagnosis or medical records. Medication histories were recorded as antihypertensive, antidiabetic, anticoagulation and lipid-lowering drugs usage.

## Quality control

Experienced professionals were invited to review and give amendments to the survey questionnaires, and their professional guidance was required. Standardized protocols and statistical methods were used, and all investigators (doctors and research technicians) underwent joint training sessions before implementation of the study.

The quality control of echocardiographic data collection followed the ‘Echocardiography laboratory standards and accreditation guidelines for adults’ recommended by ASE (Restrepo *et al.*, 2011), and the quality control of pulmonary function examination data collection followed the ‘Pulmonary Function Examination Guidelines’ recommended by the Chinese Medical Association.

### Statistical analysis

EpiData3.1 (Odense, Denmark) was used for double data input. SPSS20.0 (SPSS Inc., Chicago, IL, USA) was used for data management and analysis. Continuous variables were presented as means [95% confidence intervals (95%CI)]. Cronbach’s alpha value was calculated to assess internal reliability of MMSE and SF-36 scales. Crude differences in the proportions according to cognitive state were analysed by chi-square test. Two independent samples *t* test and the Mann-Whitney test were performed for the comparison of two independent samples. Multivariate logistic regression analysis was performed to evaluate the association between cardiopulmonary function, health-related quality of life and cognitive impairment adjusted for potential confounders. Adjusted odds ratio (AOR) and their 95%CI were reported. Variance inflation factor (VIF) was used to detect multicollinearity of variables in the model, and VIF > 10 was diagnosed as multicollinearity (Athavale *et al.*, 2021). Two-sided *P*-value <0.05 was considered statistically significant.

## Results

### Population analysis

The baseline information of 677 participants is shown in Supplementary material 3. The prevalence of cognitive impairment was 30.7% among 677 nursing home-based older population aged 80 years and over. The average age was 84.99 years (95%CI, 84.61–85.38 years). Compared to subjects with normal cognition, patients with cognitive impairment were more likely to be older, women, non-partnered, less physical activity, no history of smoking, lower BMI, no history of hypertension, dyslipidemia and coronary heart disease history (All *P* < 0.05).

One hundred and ninety-seven subjects who have effective assessment in cardiopulmonary function and HRQOL were included in the present investigation, the average age was 86.1 years (95%CI, 85.6–86.7 years), and 75% were women, average BMI was 23.91 kg/m^2^ (95%CI, 23.35–24.47 kg/m^2^). About 32.5% of the subjects were diagnosed as cognitive impairment. Demographic characteristics stratified by cognitive status are presented in Table 1. Compared to subjects with normal cognition, patients with cognitive impairment were more likely to be older, women, non-partnered, less physical activity, lower BMI, hypertensive history and no history of antihypertensive drugs (All *P* < 0.05).

**Table 1.** Demographic and health characteristics of the older nursing home residents in Shanghai, China (*N* =  197)

Variable	**Normal cognition** ***n*** **= 133**	**Cognitive impairment** ***n*** **= 64**	***P*** **-value**
**Sociodemographic characteristics**			
**Age (years)**	85.4(84.7–86.0)	87.8(86.7–88.8)	<0.001
**Gender (%)**			0.015
Men	40(30.1)	9(14.1)	
Women	93(69.9)	55(85.9)	
**Educational level (%)**			0.100
Elementary school or less	114(85.7)	60(93.8)	
Middle school or more	19(14.3)	4(6.2)	
**Marital status (%)**			0.018
Partnered	34(25.6)	7(10.9)	
Non-partnered	99(74.4)	57(89.1)	
**Physical activity (%)**			0.011
≤1 h per day	66(49.6)	44(68.8)	
>1 h per day	67(50.4)	20(31.2)	
**Smoking (%)**	19(14.3)	4(6.2)	0.100
**SBP (mmHg)**	144.55(141.21–147.89)	145.27(140.04–150.49)	0.814
**DBP (mmHg)**	71.14(69.32–72.95)	72.09(69.07–75.12)	0.655
**BMI (kg/m** ^**2**^ **)**	24.31(23.62–25.00)	22.80(21.62–23.99)	0.021
**Medical history (%)**			
Hypertension	93(69.9)	33(51.6)	0.012
Diabetes	22(16.5)	9(14.1)	0.655
Dyslipidemia	8(6.0)	1(1.6)	0.300
Coronary heart disease	29(21.8)	8(12.5)	0.117
Stroke	26(19.5)	17(26.6)	0.264
Tumour	3(2.3)	0(0.0)	0.226
**Medications (%)**			
Antihypertensive	92(69.2)	30(46.9)	0.003
Antidiabetic	20(15.0)	8(12.5)	0.633
Lipid-lowering drugs	18(13.5)	6(9.4)	0.403
Anticoagulation	40(30.1)	16(25.0)	0.460
**Cardiac function**			
Heart rate (bpm)	75.32(73.26–77.37)	76.19(73.14–79.23)	0.750
LVD (mm)	46.82(45.78–47.86)	46.75(45.42–48.08)	0.916
LVS (mm)	35.81(34.73–36.89)	36.56(35.17–37.96)	0.365
LVEF (%)	55.12(53.43–56.82)	51.67(48.86–54.48)	0.029
LVFS (%)	23.83(22.83–24.82)	21.97(20.45–23.49)	0.040
LVPW (mm)	10.05(9.90–10.21)	10.25(9.86–10.64)	0.653
IVS (mm)	9.67(9.46–9.87)	9.58(9.27–9.88)	0.460
**Respiratory function**			
VC (L)	1.62(1.53–1.70)	1.37(1.26–1.48)	0.004
MVV (L)	32.69(30.50–34.88)	27.82(25.29–30.34)	0.016
**Dimensions of the SF-36**			
PF	62.26(57.68–66.83)	46.17(39.39–52.95)	<0.001
RP	67.86(60.18–75.53)	70.31(59.19–81.43)	0.735
BP	70.02(66.76–73.28)	69.88(64.41–75.36)	0.715
GH	59.52(55.71–63.33)	54.58(49.26–59.89)	0.144
VT	79.92(76.79–83.06)	71.95(66.45–77.45)	0.014
SF	81.39(77.86–84.92)	75.78(69.59–81.97)	0.181
RE	78.70(71.90–85.50)	73.96(63.42–84.50)	0.317
MH	79.40(76.42–82.38)	75.31(70.68–79.95)	0.119
**MMSE score**	23.50(22.85–24.16)	14.44(13.49–15.39)	**<**0.001

Continuous parameters were presented as means (95% confidence intervals). Categorical variables were presented as number of participants (column percentage). BMI = body mass index; BP = body pain; DBP = diastolic blood pressure; GH = general health; IVS = the thickness of the basal interventricular septum; LVD = end-diastolic dimension of left ventricle; LVEF = left ventricular ejection fractions; LVFS = left ventricle fractional shortening; LVPW = left ventricular posterior wall; LVS = end-systolic dimension of left ventricle; MH = mental health; MMSE = Mini-Mental State Examination; MVV = maximal ventilatory volume; PF = physical functioning; RE = role limitations due to emotional problems; RP = role limitations due to physical health; SBP = systolic blood pressure; SF = social functioning; SF-36 = short form-36 scales; VC = vital capacity; VT = vitality.

Abnormal LVEF, VC and MVV detected in patients with cognitive impairment were 42.2%, 79.7% and 98.4%, respectively. When comparing to subjects with normal cognitive function, subjects with cognitive impairment showed significantly crude decline in LVEF, LVFS, VC, MVV, PF and VT (All *P* < 0.05) (Table 1).

### Sub-Domain analysis of cognitive function

**Figure 1.**
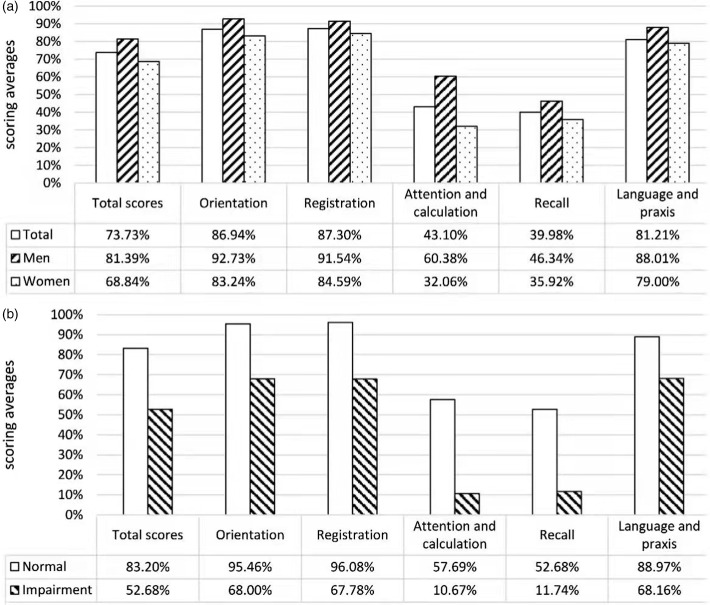
Transformed MMSE scores of five domains stratified by gender and cognitive function. The logistic regression model adjusted age and gender. The logistic regression model adjusted age and gender, marital status, physical activity and BMI. HRQOL: health-related quality of life; LVEF: left ventricular ejection fraction; LVFS: left ventricle fractional shortening; VC: vital capacity; MVV: maximal ventilator volume; PF: physical functioning; VT: vitality; BMI: body mass index; AOR, adjusted odds ratio. MMSE: Mini-Mental State Examination.

The transformed MMSE scores of domains stratified by gender and the classification of cognitive function were displayed **(**Figure 1 **)**. In the older Chinese nursing home residents, MMSE scores were clearly the lowest in attention and calculation (43.1%) and recall (40.0%). Men had higher scores in all MMSE dimensions than women. Besides, participants classified as cognitive impairment performed worse than those with normal cognition in all domains, and cognitive scores of the two groups differed most in attention and calculation (57.7% vs. 10.7%) and recall (52.7% vs. 11.7%).

### Cardiopulmonary dysfunction, HRQOL and cognitive impairment

The variables of *P* < 0.05 in the Table 1 were integrated into the multivariable logistic regression model. Multicollinearity analysis did not detect severe collinearity among these variables in the model (Supplementary material 4). Abnormal LVEF (AOR, 1.983; 95%CI, 1.034–3.805) and VC (AOR, 2.079; 95%CI, 1.069–4.041) were significantly associated with cognitive impairment after adjusting confounders including age and gender, while history of antihypertensive drugs (AOR, 0.397; 95%CI, 0.208–0.759) and higher PF (AOR, 0.982; 95%CI, 0.970–0.994) was associated with the decrease in cognitive impairment risk and this association still existed after adjusting age and gender, marital status, physical activity, and BMI (Table 2).

**Table 2.** Estimating the risk of cognitive impairment associated with factors of interest among the older nursing home residents in Shanghai, China (*N* = 197)

	Model1	Model2
Variables	AOR, 95%CI	AOR, 95%CI
Antihypertensive	0.397(0.208–0.759)	0.472(0.241–0.925)
LVEF	1.983(1.034–3.805)	1.948(0.998–3.802)
LVFS	1.172(0.614–2.238)	1.192(0.612–2.321)
VC	2.079(1.069–4.041)	1.921(0.970–3.801)
MVV	2.893(0.606–13.815)	2.995(0.600–14.954)
PF	0.982(0.970–0.994)	0.984(0.971–0.997)
VT	0.984(0.969–1.000)	0.988(0.972–1.004)

Model 1 adjusted age and gender. Model 2 adjusted age, gender, marital status, physical activity and BMI. AOR = adjusted odds ratio; BMI = body mass index; CI = confidence intervals; LVEF = left ventricular ejection fraction; LVFS = left ventricle fractional shortening; MVV = maximal ventilatory volume; PF = physical functioning; VC = vital capacity; VT = vitality.

### Analysis of five domains of cognitive function

**Figure 2.**
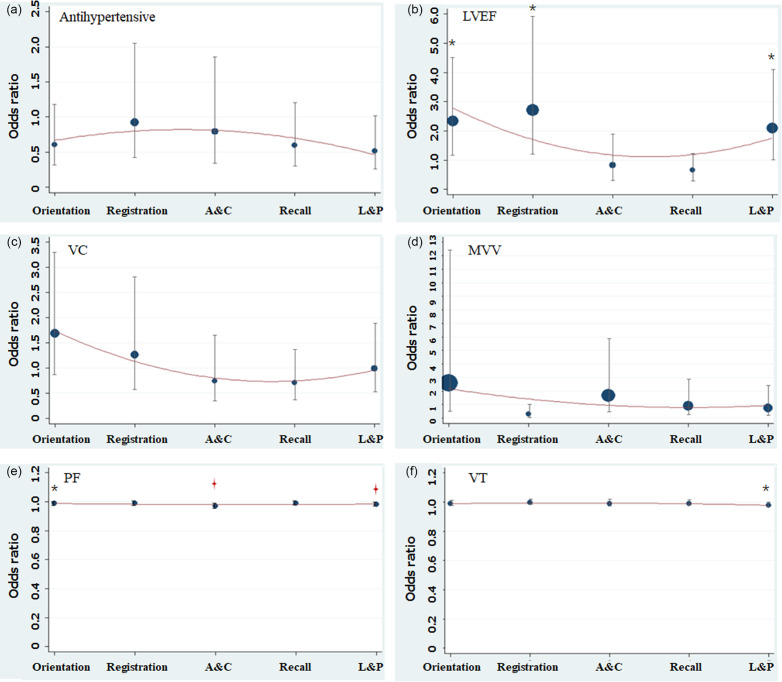
The association of cardiopulmonary function, HRQOL and five domains of cognitive function. The logistic regression model adjusted age and gender, marital status, physical activity and BMI. HRQOL: health-related quality of life; LVEF: left ventricular ejection fraction; LVFS: left ventricle fractional shortening; VC: vital capacity; MVV: maximal ventilator volume; PF: physical functioning; VT: vitality; BMI: body mass index. A&C: attention and calculation; L&P: language and praxis. *P < 0.05, ^
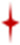
^*P* < 0.01. Size of the point represents the weight.

After adjusting age and gender, marital status, physical activity and BMI, decreasing in LVEF was related to the abnormal orientation (AOR, 2.337; 95%CI, 1.204–4.538), registration (AOR, 2.707; 95%CI, 1.237–5.924) and language and praxis (AOR, 2.085; 95%CI, 1.055–4.119). Besides, PF (AOR, 0.982; 95%CI, 0.970–0.995) and VT (AOR, 0.978; 95%CI, 0.961–0.995) were protective factors of language and praxis. Moreover, PF was associated with better attention and calculation (AOR, 0.970; 95%CI, 0.953–0.987) (Figure 2).

## Discussion

As one of the major public health concerns, cognitive impairment is not only highly prevalent, but has a negative impact on HRQOL, thus imposing a substantial socioeconomic burden (Sanford, 2017). Besides those confirmed risk factors including age, gender, marital status, personal income, physical activity, BMI and history of stroke, impaired cardiopulmonary function had already been identified as medical co-morbidity with cognitive impairment (Jia *et al.*, 2020; Duong *et al.*, 2022; Huang *et al.*, 2022). Thus, ascertain relationships between HRQOL score, cardiopulmonary function and cognitive performance are fundamental for providing evidence for the prevention and control of cognitive decline-associated disease burden.

In this study, we found that 30.7% of the older nursing home residents aged over 80 years in Shanghai were classified as cognitive impairment, which was higher than the community elderly aged over 80 years (Lv *et al.*, 2019; Jia *et al.*, 2020), suggesting it was necessary to pay close attention to the cognitive function of the elderly over 80 years old. Antihypertensive drug was found to be a protective factor of cognitive function in this study, which was consistent with some previous researches (Levi Marpillat *et al.*, 2013; Peters *et al.*, 2015; Ou *et al.*, 2020), which might be due to the fact that antihypertensive drug ameliorates or mitigates white matter lesions caused by high blood pressure (Iadecola and Gottesman, 2019).

About 42.2% of subjects with cognitive impairment had abnormal LVEF value, suggesting these residents were at a high risk of HF; 79.7% of them had abnormal VC value and might have restricted ventilation disorders; furthermore, 98.4% of cognitive impairment subjects had abnormal MVV value, indicating reduced ventilation reserve capacity. Thus, the co-prevalence of cognitive dysfunction and cardiopulmonary diseases was worthy of attention. Association between LVEF and cognitive function still remains unclear nowadays. A nonlinear U-shaped correlation between LVEF and measures of accelerated cognitive ageing was reported in one of the Framingham Heart Studies (Jefferson *et al.*, 2011). Abnormal LVEF was found to be related with cognitive impairment in our study, which was agreement with previous studies (Bossola *et al.*, 2014; Xing *et al.*, 2020; Shang *et al.*, 2022). Lower LVEF always elevates levels of catecholamine and endothelin 1 (Macrae *et al.*, 1993), and decreased cerebral blood flow then causes the damage of nerve cells, which affects cognitive function. Animal experiments also demonstrated that abnormal cardiac function can cause cognitive impairment by mediating oxidative stress (Jinawong *et al.*, 2022). Additionally, our study also found that abnormal LVEF was associated with three domains of cognitive function, including orientation, registration and language and praxis. Abnormal VC could increase the risk of cognitive impairment in older participants in this study. An explanation was that poor breathing was associated with a reduction in the lung tissues available for gas exchange; thus, it may increase the chance of hypoventilation and chronic hypoxia and hypercapnia, which may affect neurocognitive function (Giltay *et al.*, 2009); besides, elevated levels of systemic inflammation markers in patients with respiratory diseases, such as C-reactive protein, interleukin 6 and fibrinogen, might increase the risk of cognitive impairment (Su *et al.*, 2016).

Higher PF and VT scores showed significant relation with better cognitive function, which mainly shown in the orientation, attention and calculation and language and praxis domains. Physical functioning mainly assessed whether health status affects daily physiological activities. Previous studies also demonstrated that appropriate physical activities can improve cognitive function (Song and Yu, 2019; Alsubaie *et al.*, 2020), which indicated that nursing homes should focus on physical exercise of the elderly, such as setting up some exercise equipment in nursing homes. Vitality mainly evaluated individual subjective feelings about their own energy and fatigue. Prior studies also indicated that long-term fatigue and low energy had negative effect on cognitive function (Lin *et al.*, 2013; Zhang *et al.*, 2015). Physical exercise, nutrition supplementation, yoga and meditation can be effective methods to decrease fatigue and increase energy (Bower *et al.*, 2012; Ng *et al.*, 2015). Thus, more intervention trials should be performed to explore effective measures to improve PF and VT among nursing home residents.

Our study has some implications for the formulation of primary healthcare policy. Current primary health care still faces serious challenges in detection and management of cognitive impairment (Lu *et al.*, 2022). The cognitive impairment of nursing home residents over 80 is a problem that cannot be ignored. That requires healthcare providers to pay more attention to the health of nursing home residents, especially cardiopulmonary function and HRQOL. Thus, there is an urgent need to provide a range of support to facilitate healthcare providers to offer accessible, affordable and appropriate cognitive impairment-related primary healthcare services for nursing home residents, such as clinical practice guidelines, policy and financial support, and information infrastructures (Lu *et al.*, 2022).

The present study has several limitations. Firstly, cognitive impairment was identified using MMSE rather than the gold standard measures (eg, DSM-111, NINCDS-ADRDA and clinical record) and other international scales (McKhann *et al.*, 1984). Secondly, a cross-sectional study may not provide evidence of causal relationship between risk factors and cognitive impairment. A longitudinal follow-up study should be conducted to fully assess the causality. Some information such as marital status and smoking status was collected based on self-report, which could be affected by recall bias. Finally, only 197 of 677 people had qualified cardiopulmonary function tests, so selection bias may have existed.

## Conclusions

Cognitive impairment is still one of the major problems in elderly nursing home residents. Previous antihypertensive drug usage, abnormal LVEF, VC, PF and VT were associated with cognitive dysfunction. We recommend that healthcare professionals and nursing staff should increase attention to the improvement of cardiopulmonary function, PF and VT. Public health policy formulation should give more consideration to the health of the nursing home residents. Further prospective studies are needed to evaluate HRQOL and cardiopulmonary risk predictors of patients with cognitive impairment.

## Data Availability

The data of this study are available from the corresponding author upon reasonable request.

## Supplementary material

For supplementary material accompanying this paper visit http://doi.org/10.1017/S1463423623000075. click here to view supplementary material
