# Supplementary material for: Association between cardiopulmonary function, health-related quality of life and cognitive impairment among the older nursing home residents in Shanghai, China
Source: Prim Health Care Res Dev. 2023 Mar 15;24:e18. doi: 10.1017/S1463423623000075 (PMC10050953; doi:10.1017/S1463423623000075)
Supplement: Supplementary file 1 [file S1463423623000075sup001.docx]

Supplementary material：


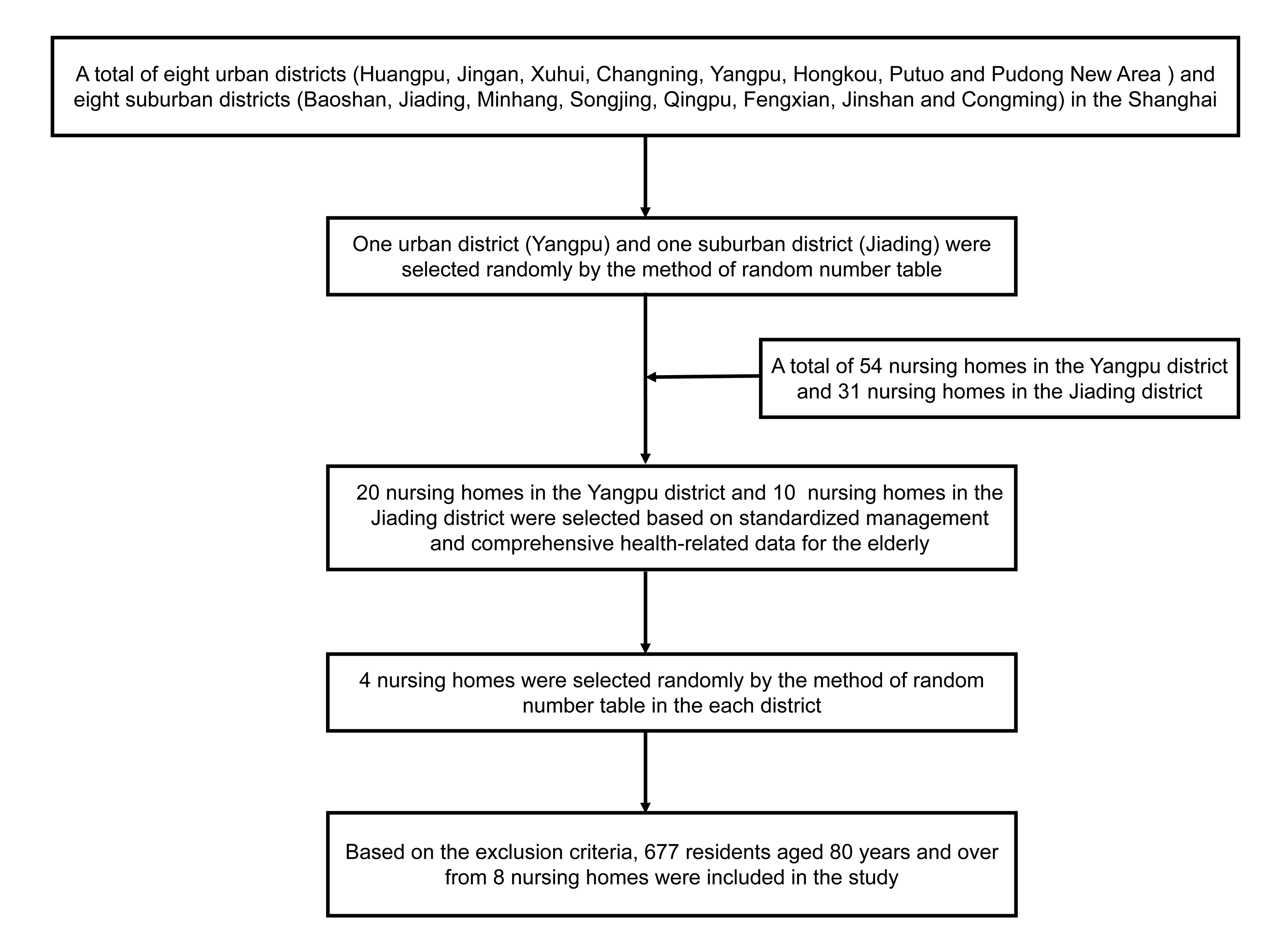


Supplementary material 1 (Figure): Sampling selection process of study population

Supplementary material 2 (Table): criteria for dividing normal and abnormal indicators

|  | **Normal** | **Abnormal** | **Note** |
| --- | --- | --- | --- |
| LVD (mm) | 35-55 | <35 or >55 |  |
| LVS (mm) | 23-40 | <23 or >40 |  |
| LVEF (%) | ≥50 | <50 |  |
| LVFS (%) | ≥25 | <25 | LVFS=[(LVD-LVS)/ LVD] *100% |
| LVPW (mm) | 7-11 | <7 or >11 |  |
| IVS (mm) | 6-12 | <6 or >12 |  |
| VC (%) | Measured value/theoretical  predicted value≥80 | Measured value/theoretical  predicted value<80 |  |
| MVV (%) | Measured value/theoretical  predicted value≥60 | Measured value/theoretical  predicted value<60 |  |

LVD: end-diastolic dimension of left ventricle; LVS: end-systolic dimension of left ventricle; LVEF: left ventricular ejection fraction; LVFS: left ventricle fractional shortening; LVPW: left ventricular posterior wall; IVS: the thickness of the basal interventricular septum; VC: vital capacity; MVV: maximal ventilatory volume.

Supplementary material 3 (Table): Demographic and health characteristics of participants with cognitive impairment and normal cognition (N=677)

| **Variable** | **Normal cognition**  **n=469** | **Cognitive impairment**  **n=208** | ***P*-value** | |
| --- | --- | --- | --- | --- |
| **Age (years)** | 84.56(84.23-84.90) | 85.96(84.96-86.96) | | <0.001 |
| **Gender (%)** |  |  | | <0.001 |
| Men | 214(45.6) | 50(24.0) | |  |
| Women | 255(54.4) | 158(76.0) | |  |
| **Marital status (%)** |  |  | | <0.001 |
| Partnered | 229(48.8) | 44(21.2) | |  |
| Non-partnered | 240(51.2) | 164(78.8) | |  |
| **Physical activity (%)** |  |  | | <0.001 |
| ≤1 hour per day | 263(56.1) | 83(39.9) | |  |
| >1 hour per day | 206(43.9) | 125(60.1) | |  |
| **Smoking (%)** | 91(19.4) | 26(12.5) | | 0.028 |
| **SBP (mmHg)** | 135.61(133.96-137.25) | 135.42(132.89-137.95) | | 0.897 |
| **DBP (mmHg)** | 76.34(75.45-77.23) | 75.70(74.30-77.10) | | 0.365 |
| **BMI (kg/m^2^)** | 23.96(23.62-24.31) | 22.85(22.32-23.78) | | <0.001 |
| **Medical history (%)** |  |  | |  |
| Hypertension | 298(63.5) | 103(49.5) | | 0.001 |
| Diabetes | 74(15.8) | 35(16.8) | | 0.732 |
| Dyslipidemia | 56(11.9) | 14(6.7) | | 0.040 |
| Coronary heart disease | 119(25.4) | 36(17.3) | | 0.021 |
| Tumor | 15(3.2) | 2(1.0) | | 0.086 |
| **Medications (%)** |  |  | |  |
| Antihypertensive | 134(28.6) | 57(27.4) | | 0.755 |
| Antidiabetic | 72(15.4) | 25(12.0) | | 0.254 |
| Lipid lowering drugs | 46(9.8) | 13(6.2) | | 0.130 |
| Anticoagulation | 106(22.6) | 53(25.5) | | 0.415 |
| **MMSE score** | 25.07(24.76-25.37) | 15.48(14.92-16.03) | | <0.001 |

Categorical variables were presented as number of participants (column percentage). Continuous variables were presented as means (95% confidence intervals). SBP: systolic blood pressure; DBP: diastolic blood pressure; BMI: Body Mass Index; MMSE: Mini Mental State Examination.

Supplementary material 4 (Table): Multicollinearity diagnosis

| **Variable** | **Tolerance** | **VIF** |
| --- | --- | --- |
| Age | 0.873 | 1.145 |
| Gender | 0.828 | 1.207 |
| Marital status | 0.825 | 1.212 |
| Physical activity | 0.813 | 1.230 |
| BMI | 0.918 | 1.090 |
| Antihypertensive | 0.891 | 1.123 |
| LVEF | 0.609 | 1.641 |
| LVFS | 0.615 | 1.625 |
| VC | 0.942 | 1.062 |
| MVV | 0.950 | 1.053 |
| PF | 0.673 | 1.487 |
| VT | 0.780 | 1.281 |

VIF: variance inflation factor; BMI: Body Mass Index; LVEF: left ventricular ejection fractions; LVFS: left ventricle fractional shortening; VC: vital capacity; MVV: maximal ventilatory volume; PF: Physical functioning; VT: Vitality.
